# Do you feel me? Autism, empathic accuracy and the double empathy problem

**DOI:** 10.1177/13623613241252320

**Published:** 2024-05-17

**Authors:** Rachael TS Cheang, Maya Skjevling, Alexandra IF Blakemore, Veena Kumari, Ignazio Puzzo

**Affiliations:** Brunel University London, UK

**Keywords:** autism spectrum disorders, double empathy problem, empathy, ASD, empathic accuracy, autism

## Abstract

Empathy deficits in autism, particularly cognitive empathy, have been a long-held, but much debated assumption. An alternative perspective challenging this deficit model is the ‘double empathy problem’, proposing that empathy difficulties are bidirectional between autistic and non-autistic people. Despite this view gaining popularity, there has been limited research examining whether non-autistic people can empathise accurately, cognitively and affectively with autistic people. Addressing this gap, 81 adults from the general population, divided into groups based on how likely they are to share personality traits common in autistic people, were examined using an empathic accuracy task, modified to include autistic and non-autistic narrators and combined with a body mapping tool. Results showed participants had significantly lower empathic accuracy scores when viewing autobiographical accounts of emotional events from autistic narrators, compared to non-autistic narrators, especially for happy and sad emotions. However, participants also experienced significantly higher intensity in the body when viewing autistic narrators compared to non-autistic narrators, especially for anger and fear emotions. These findings support the double empathy problem and have strong implications for therapeutic and interpersonal relationships with autistic people.

**Lay Abstract:**

The assumption that autistic people lack empathy, particularly imagining how others feel, has been much debated and is now being challenged by an alternative view: the ‘double empathy problem’. This suggests that non-autistic people may find it equally difficult to imagine how autistic people feel. Although this perspective is gaining popularity, research testing whether non-autistic people can accurately imagine and feel an autistic person’s emotions is still limited. Our study used video clips of autistic and non-autistic people recounting emotional events to test if participants from the general population could: track the intensity of the narrators’ emotions; name and feel the same emotion; match where the narrator felt the emotion and indicate how intensely they felt the emotion using a body map. Our results show that participants found it significantly harder to track autistic narrators’ emotions compared to non-autistic narrator’s emotions, especially when viewing clips of narrators feeling happy and sad. We also found that participants felt emotions more intensely in the body when viewing clips of autistic narrators compared to non-autistic narrators, especially when describing anger and fear. These findings support the double empathy problem and have strong implications for therapeutic and interpersonal relationships with autistic people.

Autistic people have long been perceived by professionals and society as having a lack of empathy (Baron-Cohen et al., 2001). However, research has begun to critically evaluate the term empathy when exploring autistic empathy, as inconsistencies in defining terms often result in confusion when interpreting research findings and more importantly misunderstandings about autistic people within the general population and among professionals (Blair, 2008; Bollen, 2023; Cuff et al., 2016; Fletcher-Watson & Bird, 2020; Harrison et al., 2022). For autistic people, such misunderstandings can be painful and dehumanising, contributing to poor self-esteem and mental well-being (Cohen-Rottenberg, 2011; Nicolaidis et al., 2019). An alternative emerging view in autism research, challenging the notion of an empathy deficit altogether, proposes, instead, a ‘double empathy problem’ (Milton et al., 2018). This suggests that when people with very different world views interact, they struggle to empathise with each other, thus re-framing the empathy issue as one of ‘reciprocity and mutuality’ (Milton, 2012).

Empathy, defined broadly as ‘an emotional reaction in an observer to the affective state of another individual’ (Blair, 2005), is understood to be multidimensional, comprising of at least two components: affective empathy (AffEmp) and cognitive empathy (CogEmp) (Davis, 1983; Dvash & Shamay-Tsoory, 2014; Eisenberg & Miller, 1987; Mazza et al., 2014; Zaki et al., 2008). While research suggests autistic people show differences in their ability to empathise (Baron-Cohen et al., 1997, 1985, 2001; Capps et al., 1993; Happé, 1994; Harrison et al., 2022; Hill & Frith, 2003; Jones et al., 2010; Leppanen et al., 2018; Magnée et al., 2007; Mazza et al., 2014; Rogers et al., 2007), evidence that autistic people lack theory of mind (ToM), a component of CogEmp, appears more universally accepted. A systematic review revealed that overall, autistic people performed significantly less well than non-autistic people, with large effect sizes on cognitive ToM tasks (Leppanen et al., 2018). However, numerous empirical flaws have been highlighted in these studies (Gernsbacher & Yergeau, 2019). Studies examining differences in AffEmp are more inconsistent: Results show autistic people have similar (Mazza et al., 2014; Mul et al., 2018; Rogers et al., 2007), increased (Capps et al., 1993; Magnée et al., 2007) or decreased (Baron-Cohen & Wheelwright, 2004; Lombardo et al., 2007) AffEmp compared to non-autistic people.

While affective and cognitive components are often measured concurrently in self-report questionnaires (Baron-Cohen & Wheelwright, 2004; Davis, 1983), objective performance tasks typically focus on only CogEmp or AffEmp and use static images, failing to capture the more nuanced, complex nature of empathy (Rum & Perry, 2020). Empathic accuracy (EmpAcc) tasks attempt to address this by measuring the ability to accurately infer thoughts and feelings of another using video clips, which are more dynamic and ecologically valid (Ickes et al., 1990; Mackes et al., 2018; Mckenzie et al., 2022; Zaki et al., 2008). Synthesised findings from a scoping review revealed autistic people and non-autistic people with personality traits similar to those assumed common among the autistic community, exhibit deficits in EmpAcc (Rum & Perry, 2020).

Nonetheless, a recent study employing a modified EmpAcc task, including additional measures of CogEmp and AffEmp, showed autistic adults only demonstrated deficits in EmpAcc for anger compared to non-autistic adults. Furthermore, the autistic people studied showed no deficits in CogEmp or AffEmp on the EmpAcc task, despite reporting deficits in trait CogEmp and AffEmp on a self-report measure (Mckenzie et al., 2022). However, CogEmp and AffEmp on the EmpAcc task were measured by asking which emotion the participant thought the narrator was feeling and how they felt watching the clip. This yielded results that were either ‘right’ or ‘wrong’, generating over-simplified measurements of AffEmp and CogEmp. The authors noted this limitation suggesting that more nuanced measures of these constructs were required (Mckenzie et al., 2022). Santiesteban et al. (2021) described the CogEmp and AffEmp measures in the EmpAcc task as ‘offline’ measures (i.e. measured retrospectively) and the EmpAcc measurement itself as an ‘online’ measure and so extended the EmpAcc task to include continuous self-ratings. Results from this study showed that autistic people only showed reduced CogEmp and AffEmp compared to non-autistic people when measured offline, but not online; however, this task only asked participants to rate the intensity of emotion, rather than identify which emotion was being felt, which in this study was either sad or neutral only (Santiesteban et al., 2021).

## The double empathy problem

The ‘double empathy problem’ was proposed based on Milton’s own experience, anecdotal accounts and limited amounts of qualitative data (Milton et al., 2022). However, an increasing number of studies have explored this concept. Crompton et al. (2020) showed that information passed through chains of people with similar neurotypes (autistic or non-autistic) was more accurate than through chains of mixed neurotypes. Moreover, participant rapport ratings were significantly lower for mixed chains. Similarly, studies report that autistic people feel less stressed interacting with other autistic people (Camus et al., 2022), prefer within-neurotype interactions (Chen et al., 2021), create mutual understanding during interactions with other autistic people (Heasman & Gillespie, 2019) and display fewer autistic traits when interacting with other autistic people (Gernsbacher et al., 2017). Furthermore, interpersonal similarity of autistic traits within the general population is associated with increased friendship quality (Bolis et al., 2021).

There has been some exploration of how well non-autistic people interpret the emotional expressions and mental states of autistic people. Brewer et al. (2016) reported non-autistic observers as less able to recognise photographed autistic emotional expressions compared to non-autistic expressions. Similarly, they found autistic observers equally poor at recognising autistic emotional expressions. Sheppard et al. (2016) conducted a series of studies where non-autistic participants were shown short, muted videos in which autistic and non-autistic targets responded to one of four scenarios (joke, waiting, story and compliments). Participants had to decide which scenario provoked which response, score how expressive targets were and describe the response. Although participants found autistic targets as expressive as non-autistic targets in 75% of scenarios, they found it significantly more difficult to identify which scenario autistic targets were responding to compared to non-autistic targets (Sheppard et al., 2016), perhaps demonstrating their struggle to represent mental states of autistic individuals. Edey et al. (2016) used animations of triangles generated by autistic and non-autistic people to depict mental state interactions (coaxing, mocking, seducing and surprising) seeing if autistic and non-autistic people could infer correct mental states from both groups. Researchers found that non-autistic perceivers had enhanced ability to infer correct mental states on non-autistic animations compared to autistic animations, but autistic perceivers showed no difference in identifying mental states of animations created by autistic and non-autistic participants.

Although the above studies show that (1) non-autistic people struggle to represent mental states of autistic people and (2) non-autistic people struggle to recognise autistic people’s emotions from photographed facial expressions, the stimuli used did not adequately capture the dynamic, nuanced and complex expression of emotion. Furthermore, these studies only focused on CogEmp and not AffEmp, an embodied empathic response to another’s emotions. The present study aims to fill these gaps, undoubtedly important in therapeutic and interpersonal relationships. Camm-Crosbie et al. (2019) developed an online survey exploring experiences of autistic adults receiving treatment and support for mental health problems. Three underlying themes emerged: (1) many autistic adults, perceived as ‘coping’, were dismissed for treatment; (2) many professionals lacked understanding of how autistic people communicate, socially interact and crucially show and express feelings and emotions; and (3) lack of appropriate support contributed to poor mental health outcomes, self-injury and suicidality in autistic people. It is, therefore, crucial to understand whether non-autistic people can accurately empathise with autistic people’s emotions.

## The current study

Primarily, this study aimed to understand whether people from the general population (not considered autistic), can accurately, cognitively and affectively empathise with autistic and non-autistic narrator’s emotions. Based on previous research showing non-autistic people struggling to represent mental states of autistic people and that autistic people feel their emotions are misunderstood (Camm-Crosbie et al., 2019; Sheppard et al., 2016), we expected that participants from the general population would struggle to empathise accurately, cognitively and affectively, with the emotions of autistic narrators. Second, we aimed to test whether individuals from the general population with Autism-Spectrum Quotient (AQ) scores closer to those found in autistic people empathise more accurately, cognitively and affectively with autistic narrators than with non-autistic narrators compared to those with a greater mismatch in AQ scores. Although the AQ has been used previously as a measure of ‘autistic traits’ in the general population, it should be noted that autistic traits are not homogeneous among autistic people and assumptions of what constitutes low, medium, or high autistic traits are subjective judgements. Nonetheless, given that there is some evidence to suggest that higher interpersonal similarity of autistic traits in the general population is associated with higher measures of closeness, acceptance and help (Bolis et al., 2021), we predicted that participants from the general population with higher AQ scores would empathise more accurately, cognitively and affectively with autistic narrators compared to non-autistic narrators than non-autistic participants with lower AQ scores. Our novel approach combined a modified version of the EmpAcc task (N. A. Martin-Key et al., 2017) manipulating the narrator type to include autistic and non-autistic narrators with a modified version of the emBODY tool (Nummenmaa et al., 2014), which maps bodily sensations associated with emotions, obtaining a more refined measure of AffEmp. The emBODY tool has been used in previous research to examine how body maps of emotion are related to trait measures of empathy (Sachs et al., 2019).

## Method

### Participants

Eighty-five adults from the general population were recruited through the Brunel University Intranet and various social media platforms. An a priori power analysis using G-Power suggested a sample size of 87 based on a repeated measures analysis of variance (ANOVA) with three groups and 10 performance measures, detecting an effect size of 0.15 and significance level of 0.05% and 95% power. Participants were eligible for the study if they could travel to Brunel University London for the study; were aged 18–85 years; were fluent in English; had no known history of psychosis, substance abuse or a traumatic brain injury; and had no genetic disorders affecting brain function or intellectual disability. The participants did not disclose whether they were autistic or not, but the mean AQ score fell below the clinical cut off and so participants were considered non-autistic. Ethical approval was granted by the College of Health, Medicine and Life Sciences Research Ethics Committee, Brunel University London (reference: 32839-A-May/2022-39560-2). All participants provided written consent and received £10 (Amazon voucher) or course credits for their participation.

### Measure of autistic traits

Autistic traits in our sample from the general population were measured using the 50-item self-report AQ (Baron-Cohen et al., 2001). The scale has good psychometric properties, with normally distributed AQ sum scores (score range 0–50, with ⩾29 indicating clinically significant autism traits) in the general population (Hurst et al., 2007). The scale had a high level of internal consistency in the current sample (Cronbach’s α = 0.848).

### Behavioural tasks

#### The empathic accuracy task

**Figure 1.**
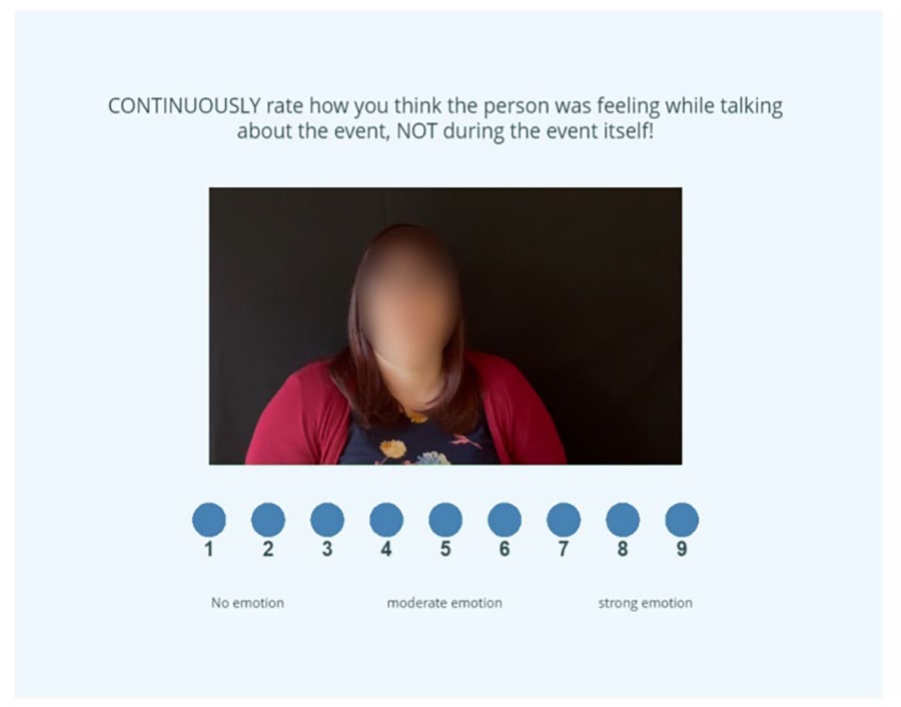
Example of continuous rating during the empathic accuracy task. *Note.* Participants were asked to rate on a 9-point scale how emotional they thought the narrator was feeling while recounting an autobiographical event. 0 indicated no emotion, 9 indicated strong emotion.

The EmpAcc task was first designed to measure perceivers’ ability to accurately assess targets’ emotions (Zaki et al., 2008) and was subsequently modified by Martin-Key et al. (2017) to include videos depicting primary emotions instead of undifferentiated positively and negatively valenced stimuli. We further modified the task to include autistic narrators and non-autistic narrators to allow the double empathy problem to be examined (description of task construction in Supplementary Material). During the EmpAcc task, participants were shown 10 pseudo-randomised video clips of narrators giving autobiographical accounts of situations where they felt one of four primary emotions (anger, fear, happiness and sadness) and a neutral event. The participants were instructed to continually rate the intensity of the emotion on the 9-point scale while viewing a training clip (see Figure 1). It was emphasised that the participant should rate the intensity of the emotion being experienced by the narrator while they were speaking about the event, not the intensity felt during the event itself. An EmpAcc score was formed by correlating the participants’ continuous ratings of emotional intensity to the narrators’. The participants were then asked to name the emotion the narrator was experiencing while speaking (options: happy, sad, frightened, angry, surprised and no emotion) (CogEmp), as well as the emotion they felt during the task (same six options) (AffEmp).

#### The emBODY task

**Figure 2.**
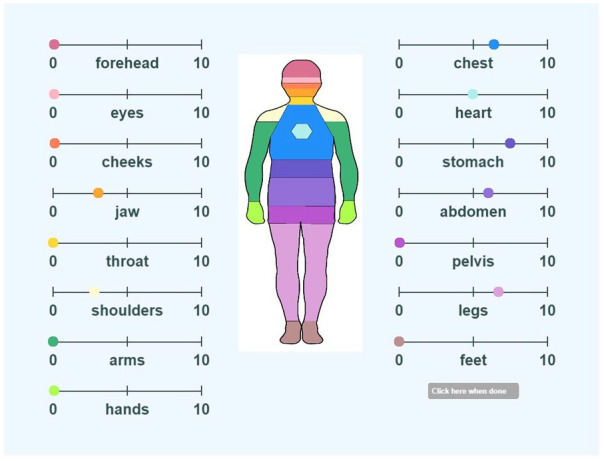
Example of body mapping tool. *Note.* After watching each video clip, participants were asked to identify on the body map where they felt sensation and at what intensity on a 10-point scale. 0 indicated no sensation, 10 indicated intense sensation.

The emBODY task is a computer-based, topographical self-report task developed to map bodily sensations associated with different emotions (Nummenmaa et al., 2014). Different emotions were consistently associated with statistically separable bodily sensation maps. The present study modified the maps so that 15 discrete areas of the body (top head, eyes, cheeks, jaw, throat, shoulders, arms, hands, chest, heart, stomach, abdomen, pelvis, legs and feet) could be rated for intensity of feeling on a scale of 0–10, 0 being no sensation and 10 being intense sensation (see Figure 2). The total number of body parts that matched the narrators’ body parts was calculated (BodyEmp); a match in a body part was given a score of 1 if the participant and the narrator both had a felt sense of any intensity or both had no felt sense in that body part. A maximum score of 15 meant a total match. The participants’ average intensity rating across all body parts (ParInt) was also calculated.

### Procedure

The participants were given a participant information sheet stating the aims of the research and a consent form to complete. Demographic information (age, gender and ethnic origin) was collected; however, data on socioeconomic status and educational attainment levels were not recorded. The participants completed the AQ self-report measure, followed by the two behavioural tasks. The participants were unaware of the diagnostic status of the narrators during the task but were debriefed after completion of the study.

### Analysis

Collected data were analysed using SPSS v.29 software. The mean AQ score was calculated, and the sample split into three groups using quartiles: Low-AQ (lower quartile, AQ < 13), High-AQ (upper quartile, AQ > 24) and Medium-AQ (interquartile range, AQ = 13–24). Four participants were excluded as they completed <75% of the behavioural task, leaving 81 participants (21 in Low-AQ group, 38 in Medium-AQ group, and 22 in High-AQ group). Possible group differences in demographic data were examined using a one-way ANOVA (age) or chi-square tests (gender and ethnicity).

To test the double empathy hypothesis, three-way mixed ANOVAs with one between-subjects factor (Group: Low-AQ/Medium-AQ/High-AQ) and two within-subject factors (Narrator-Type: autistic/non-autistic, Emotion: angry/sadness/fear/happiness/neutral) were run to determine the effect of different Narrator-Types and different Emotions on EmpAcc, BodyEmp and ParInt between participants with High-AQ, Medium-AQ and Low-AQ. There were multiple outliers in the data, as assessed by inspection of a boxplot, but were kept in the analysis because they did not materially affect the results as assessed by a comparison of the results with and without the outliers. EmpAcc, BodyEmp and ParInt scores were not normally distributed, as assessed by Shapiro-Wilk’s test (*p* > 0.05). As ANOVAs are fairly robust against deviations from normality and the sample size was fairly large, we continued without transformation of the data. However, as the underlying data had a skewed distribution, we used the median version of Levene’s test for equality of variances as suggested by Brown and Forsythe (1974), which revealed homogeneity of variances (*p* > 0.05). For the three-way interaction effect, Mauchly’s test of sphericity indicated that the assumption of sphericity had been violated for EmpAcc, χ^2^(9) = 52.10, *p* < 0.001; BodyEmp, χ^2^(9) = 136.38, *p* < 0.001; and ParInt, χ^2^(9) = 65.01, *p* < 0.001. The estimated epsilons (ε) were 0.713, 0.524 and 0.753, respectively; therefore, as suggested by Maxwell and Delaney (2004), the Greenhouse-Geisser correction was used.

Two-way mixed ANOVAs with one between-subjects factor and one within-subjects factor were also performed to determine the effect of different Narrator-Types on CogEmp and AffEmp, between participants with High-AQ, Medium-AQ and Low-AQ. There were no outliers, as assessed by examination of studentised residuals for values greater than ±3. CogEmp and AffEmp scores were not normally distributed, as assessed by Shapiro-Wilk’s test (*p* > 0.05). As above, we decided to continue without transformation of the data: The median version of Levene’s test for equality of variances for use, as suggested by Brown and Forsythe (1974), was homogeneity of variances (*p* > 0.05). There was homogeneity of covariances, as assessed by Box’s test of equality of covariance matrices: for CogEmp *p* = 0.305 and AffEmp *p* = 0.372.

### Community involvement

The research team included autistic (first author) and non-autistic researchers. The study design and research questions were formulated by the autistic researcher. Furthermore, the video clips used in the EmpAcc task were filmed with two autistic and two non-autistic narrators. There was no further community involvement in the design of this study.

## Results

### Demographics

There were no Group differences in age, *F*(2,77) = 1.61, *p* = 0.207; gender, χ^2^(4) = 4.12, *p* = 0.390; or ethnicity, χ^2^(12) = 9.85, *p* = 0.629 (Table 1).

**Table 1.** Demographic characteristics of the sample, classified by group.

	Low-AQ < 13 (*n* = 21^a^)	Medium-AQ = 13–24 (*n* = 38)	High-AQ > 24 (*n* = 22)	*p* value
Age	32.6 (13.7)	27.3 (10.5)	32.2 (15.1)	0.207
Gender				0.390
Female	12 (22.2)	27 (50)	15 (27.8)	
Male	9 (34.6)	11 (42.3)	6 (23.1)	
Other	0 (0)	0 (0)	1 (100)	
Ethnicity				0.629
Arab	1 (16.7)	3 (50)	2 (33.3)	
Asian	5 (25)	11 (55)	4 (20)	
Black	0 (0)	3 (75)	1 (25)	
Latina	0 (0)	1 (100)	0 (0)	
Mixed	3 (60)	1 (20)	1 (20)	
Persian	0 (0)	0 (0)	1 (100)	
White	12 (27.3)	19 (43.2)	13 (29.5)	
AQ score	9.1 (2.1)	18.9 (3.7)	28.9 (3.2)	

*Note.* Mean (std. dev) is given for age, all other values show numbers of participants (%).

a *n* = 20 for age only as data for one participant was missing.

## Empathic accuracy (EmpAcc)

**Figure 3.**
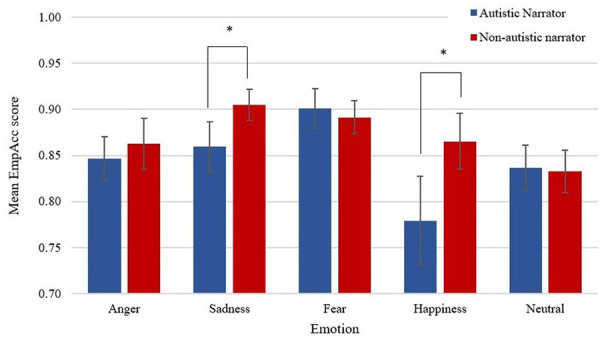
Mean empathic accuracy scores by narrator-type and emotion. *Note.* Mean empathic accuracy scores by Narrator-Type and Emption (error bars show 95% confidence interval). *p < 0.01.

There was a significant main effect of Narrator-Type, *F*(1, 73) = 8.95, *p* = 0.004, partial η^2^ = 0.109, with participants across the sample showing lower EmpAcc when viewing clips of autistic narrators than clips of non-autistic narrators. There was a significant two-way interaction between Narrator-Type and Emotion, *F*(2.85, 208.17) = 4.592, *p* = 0.005, ε = 0.713, partial η^2^ = 0.059 (see Table 2 and Figure 3). Therefore, simple main effects were run with Bonferroni adjustment applied. EmpAcc was significantly different for non-autistic narrators (*M* = 0.91, *SD* = 0.07) compared to autistic narrators (*M* = 0.86, *SD* = 0.12) for sadness, *F*(1, 73) = 7.77, *p* = 0.007, partial η^2^ = 0.096, a mean difference of –0.043, 95% confidence interval (CI) = [–0.074, –0.012] and for non-autistic narrators (*M* = 0.87, *SD* = 0.13) compared to autistic narrators (*M* = 0.78, *SD* = 0.21) for happiness, *F*(1, 73) = 9.28, *p* = 0.003, partial η^2^ = 0.113, a mean difference of –0.09, 95% CI = [–0.15, –0.03]. EmpAcc was not significantly different for non-autistic narrators compared to autistic narrators for anger, fear or the neutral condition. There was no significant three-way interaction between Narrator-Type, Emotion and Group. There were no significant two-way interactions between Narrator-Type and Group or Emotion and Group (see Supplementary Material for non-significant results).

**Table 2.** ANOVA statistics for behavioural task performance.

Variable	*df_Num_*		*df_Den_*	ε	*F*	*p*	η^2^
EmpAcc							
Narrator		1	73		8.95**	0.004	0.109
Emotion		2.76	201.18	0.689	9.61***	<0.001	0.116
Group		2	73		0.21	0.811	0.006
Narrator × Group		2	73	0.713	0.21	0.808	0.006
Emotion × Group		5.51	201.18	0.689	0.20	0.971	0.005
Narrator × Emotion		2.85	208.17	0.713	4.59**	0.005	0.059
Narrator × Emotion × Group		5.70	208.17	0.713	0.61	0.717	0.016
BodyEmp							
Narrator		1	78		0.64	0.428	0.008
Emotion		2.06	160.47	0.514	5.51**	0.004	0.066
Group		2	78		0.02	0.985	0.000
Narrator × Group		2	78	0.524	0.78	0.462	0.020
Emotion × Group		4.12	160.47	0.514	0.21	0.937	0.005
Narrator × Emotion		2.10	163.45	0.524	0.39	0.690	0.005
Narrator × Emotion × Group		4.11	163.45	0.524	0.90	0.469	0.023
ParInt							
Narrator		1	78		17.19***	<0.001	0.181
Emotion		3.28	255.72	0.820	16.96***	<0.001	0.179
Group		2	78		1.958	0.148	0.048
Narrator × Group		2	78	0.753	1.088	0.342	0.027
Emotion × Group		6.56	255.72	0.820	1.114	0.355	0.028
Narrator × Emotion		3.01	234.86	0.753	3.576*	0.015	0.044
Narrator × Emotion × Group		6.02	234.86	0.753	0.478	0.825	0.012
CogEmp							
Narrator		1	78		0.17	0.685	0.002
Group		2	78		2.97	0.057	0.071
Narrator × Group		2	78		0.27	0.764	0.007
AffEmp							
Narrator		1	78		0.16	0.687	0.002
Group		2	78		4.49*	0.014	0.103
Narrator × Group		2	78		0.14	0.869	0.004

*Note. df_Num_* indicates degrees of freedom numerator. *df_Den_* indicates degrees of freedom denominator. ε indicates Greenhouse-Geisser multiplier for degrees of freedom, *p* values and degrees of freedom incorporate this correction.

* *p* < 0.05. **p < 0.01. ***p < 0.001.

## Participant interoceptive intensity (ParInt)

**Figure 4.**
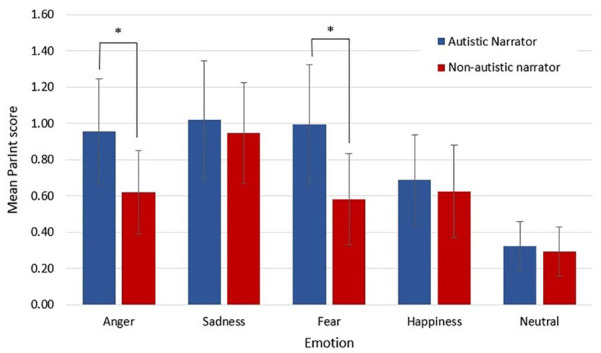
Mean participant intensity score by narrator-type and emotion. *Note.* Mean participant intensity scores by Narrator-Type and Emotion (error bars show 95% confidence interval). *p < 0.001.

There was a significant main effect of Narrator-Type, *F*(1, 78) = 17.19, *p* < 0.001, partial η^2^ = 0.181, with participants across the sample showing higher ParInt when viewing clips of autistic narrators than clips of non-autistic narrators. There was a significant two-way interaction between Narrator-Type and Emotion, *F*(3.01, 234.86) = 3.576, *p* = 0.002, ε = 0.753, partial η^2^ = 0.044 (see Table 2 and Figure 4). Therefore, simple main effects were run with Bonferroni adjustment applied. ParInt was significantly lower for non-autistic narrators (*M* = 0.59, *SD* = 0.98) compared to autistic narrators (*M* = 0.91, *SD* = 1.25) for anger, *F*(1, 78) = 21.19, *p* < 0.001, partial η^2^ = 0.214, a mean difference of 0.33, 95% CI = [0.19, 0.47] and for non-autistic narrators (*M* = 0.55, *SD* = 1.08) compared to autistic narrators (*M* = 0.95, *SD* = 1.40) for fear, *F*(1, 78) = 11.814, *p* < 0.001, partial η^2^ = 0.132, a mean difference of 0.40, 95% CI = [0.17, 0.64]. ParInt was not significantly different for non-autistic narrators compared to autistic narrators for sadness, happiness or the neutral condition. There was no significant three-way interaction between Narrator-Type, Emotion and Group. There were no significant two-way interactions between Narrator-Type and Group or Emotion and Group (see Supplementary Material for non-significant results).

## Interoceptive empathy (BodyEmp)

There was no significant main effect of Narrator-Type; participants across the sample did not differ in BodyEmp when viewing clips of autistic and non-autistic narrators. There was no significant three-way interaction involving Narrator-Type, Emotion and Group. There were also no significant two-way interactions between Narrator-Type and Group, Emotion and Group, or Narrator-Type and Emotion (see Supplementary Material for non-significant results).

## Cognitive empathy (CogEmp)

**Figure 5.**
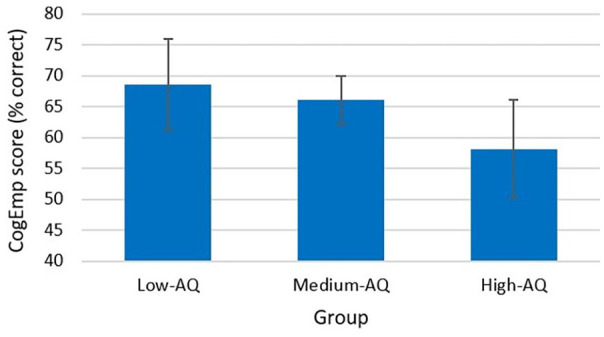
Cognitive empathy score by group. *Note.* Cognitive empathy scores are calculated as a percentage of correctly identified emotions (error bars show 95% confidence interval).

The main effect of Group was approaching significance, *F*(2, 78) = 2.97, *p* = 0.057, partial η^2^ = 0.07 (High-AQ Group, *M* = 58.2, *SD* = 17.9; Medium-AQ Group, *M* = 66.1, *SD* = 12.0; Low-AQ Group, *M* = 68.6, *SD* = 16.2) (see Table 2 and Figure 5). Post hoc analysis with a Bonferroni adjustment revealed that the mean difference in CogEmp between participants in the Low-AQ Group and the High-AQ Group (10.39, 95% CI = [–0.73, 21.50]) was approaching statistical significance, *p* = 0.075. The main effect of Narrator-Type was not significantly different for CogEmp, *F*(1, 78) = 0.17, *p* = 0.685, partial η^2^ = 0.002. Participants across the sample did not differ in CogEmp when viewing clips of autistic narrators compared to clips of non-autistic narrators. There was no significant interaction between Narrator-Type and Group, *F*(2, 78) = 0.27, *p* = 0.764, partial η^2^ = 0.007 (see Table 2).

## Affective empathy (AffEmp)

**Figure 6.**
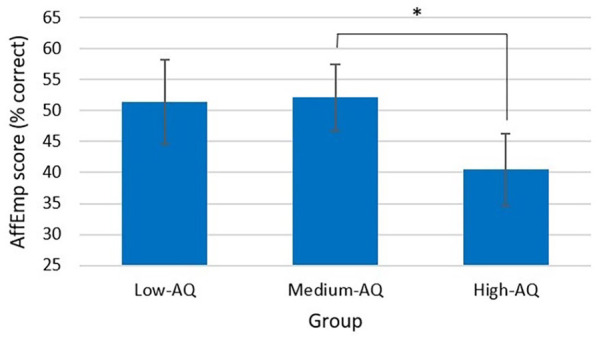
Affective empathy score by group. *Note.* Affective empathy scores are calculated as a percentage of correctly identified emotions (error bars show 95% confidence interval).

There was a significant difference in AffEmp between participants with High-AQ (*M* = 40.5, *SD* = 13.3), Medium-AQ (*M* = 52.1, *SD* = 16.5) and Low-AQ (*M* = 51.4, *SD* = 14.9), *F*(2, 78) = 4.49, *p* = 0.014, partial η^2^ = 0.103 (see Table 2 and Figure 6). Post hoc analysis with a Bonferroni adjustment revealed that the mean difference in AffEmp between participants in the Medium-AQ Group and the High-AQ Group (11.65, 95% CI = [1.64, 21.66]) was significant (*p* = 0.017) and the mean difference in AffEmp between participants in the Low-AQ Group and the High-AQ Group (10.97, 95% CI = [–0.42, 22.37]) was approaching statistical significance, *p* = 0.063. The main effect of Narrator-Type was not significant for AffEmp, *F*(1, 78) = 0.16, *p* = 0.687, partial η^2^ = 0.002. There was no significant interaction between Narrator-Type and Group, *F*(2, 78) = 0.14, *p* = 0.869, partial η^2^ = 0.004 (see Table 2).

## Discussion

This novel study investigated non-autistic participants’ ability to continuously track changes in emotional intensity (EmpAcc) in autistic and non-autistic narrators while recounting autobiographical emotional events. Participants were asked to identify the emotion they thought narrators were feeling (CogEmp), label the emotion they felt while watching the clips (AffEmp) and indicate on a body map where they were feeling the emotion (BodyEmp) and at what intensity they felt the emotion (ParInt).

Consistent with our hypothesis, we found our sample of non-autistic participants had significantly less EmpAcc with the emotions of autistic narrators compared to non-autistic narrators – an effect primarily driven by narrators describing happy or sad events. Inversely, non-autistic participants felt the emotions of autistic narrators significantly more intensely in the body compared to non-autistic narrators and that this effect was primarily driven by narrators describing events where they felt anger or fear. Despite emotions being felt more intensely in the body, the locations of where non-autistic participants and narrators felt the emotions matched, regardless of whether narrators were autistic or not.

Although no previous studies have measured EmpAcc, ParInt or BodyEmp in non-autistic participants while viewing clips of autistic and non-autistic narrators, these findings are comparable with those reported by Brewer et al. (2016), who found non-autistic participants struggle to recognise emotions of autistic participants from photographed facial expressions; by Sheppard et al. (2016) and Edey et al. (2016) who found non-autistic participants struggle to represent mental states of autistic compared to non-autistic individuals; and by Faso et al. (2015) who found autistic expressions were rated as more intense than non-autistic expressions.

One possible explanation for this finding could be that autistic individuals express emotion differently from non-autistic individuals. Zaki et al. (2008) found expressivity of the narrator a significant predictor of EmpAcc. Although early accounts of autism describe autistic individuals as having flat affect (Kanner, 1943), recent research suggests they express emotions atypically (Brewer et al., 2016; Faso et al., 2015; Volker et al., 2009). Some research demonstrates emotion recognition was linked to atypical facial and vocal expression (Brewer et al., 2016; Macdonald et al., 1989), whereas other research showed facial and vocal expressiveness was more awkward in autistic individuals, yet expressions were as recognisable as non-autistic individuals’ expressions (Grossman et al., 2013). Moreover, Sheppard et al. (2016) found that although non-autistic participants struggled to assign correct mental states to autistic targets, they were rated as similarly expressive to the non-autistic targets. Inconsistencies in these findings have been linked to differences in sex, age, intelligence and context, which all play a role in how expressive autistic samples appear (Begeer et al., 2008; Capps et al., 1993; Faso et al., 2015). For example, Capps et al. (1993) found autistic children with intellectual disability less expressive than those without and Faso et al. (2015) found emotional expressions produced by autistic females were less intense and less identifiable, but more natural than those by autistic males.

Another possibility for inconsistency could be the presence or absence of alexithymia, which co-occurs in 50% of autistic people (Kinnaird et al., 2019). One study found judges were less able to decode spontaneous facial expressions of participants with alexithymia compared to those without alexithymia and rated their expressions as less intense (McDonald & Prkachin, 1990). However, when the same participants were asked to pose facial emotions, alexithymic participants’ and non-alexithymic participants’ expressions were judged as accurately, but alexithymic participants’ expressions were significantly less intense than the non-alexithymic participants’ expressions for anger and happiness only. This finding may suggest that alexithymic people can force expression, but it comes across as less natural and, therefore, less intense. Conversely, Faso et al. (2015) reported that autistic expressions were judged more accurately than non-autistic expressions, during both posed and evoked expressions of anger, but less accurately than non-autistic expressions during posed expressions of happiness. Furthermore, aligned with our findings, autistic expressions were rated as significantly more intense than non-autistic expressions during the evoked condition for anger and happiness and the posed condition for anger and fear, but significantly less intense for sadness in the evoked condition only. Faso et al. (2015) did not check whether autistic participants also had alexithymia. Future research could examine whether alexithymia is a significant predictor of emotion expressivity in autistic people.

Our data similarly showed the main effect of Narrator on EmpAcc was driven by specific emotions, namely, happiness and sadness, but not anger or fear. This is consistent with previous findings; for example, Volker et al. (2009) reported that non-autistic raters were significantly less able to correctly identify the target emotion as happy or sad for autistic children compared to non-autistic children, although when controlling for IQ, only the difference between groups for sadness remained. Similarly, both Faso et al. (2015) and Brewer et al. (2016) found happiness the hardest emotional expression to recognise in autistic adults compared to non-autistic adults. Yet, our finding also shows that while EmpAcc scores were similar for autistic and non-autistic narrators for anger and fear, autistic narrators evoked more intense feelings in the body for these emotions than non-autistic narrators. Feeling anger and fear more intensely in the body could have led participants to track the autistic narrators’ emotions more accurately. Sachs et al. (2019), for example, found a correlation between trait perspective taking scores and the degree of overlap between self and other body maps.

These results have important implications for both therapeutic and interpersonal relationships. For example, non-autistic people finding it difficult to understand when an autistic person is happy, may fail to celebrate life’s joys with them. Equally, if non-autistic people cannot recognise when an autistic person is upset, they will fail to offer appropriate comfort and support. The lack of sharing in one’s happiness and comforting in one’s sadness may lead to autistic people to feel isolated, frustrated and angry. Furthermore, autistic people’s anger and fear being felt more intensely during interactions may lead to autistic people being misperceived as ‘less warm’ or perceived ‘less favourably’ than non-autistic people, as found in studies examining real-world social interactions between autistic and non-autistic adults (Morrison et al., 2020; Sasson et al., 2017). Faso et al. (2015) also posited that misinterpreting autistic people as angrier than they are can negatively impact social interaction. Being misinterpreted encourages autistic people to camouflage to feel socially accepted, which can lead to burnout (Grace et al., 2022; Raymaker et al., 2020). Moreover, feeling unsupported when distressed can lead to feelings of isolation. Loneliness has been shown to contribute to low self-esteem and extremely high levels of depression, self-harm and suicidality in autistic people (Grace et al., 2022; Hedley et al., 2018; Mazurek, 2014; Moseley et al., 2021).

We further explored whether participants with higher AQ scores would empathise more accurately, cognitively and affectively with autistic narrators compared to non-autistic narrators than participants with lower AQ scores. We did not find any significant interactions between Narrator-Type and Group for EmpAcc, BodyEmp, ParInt, CogEmp or AffEmp, meaning participants from all three groups empathised as accurately and equally well, cognitively and affectively, with autistic compared to non-autistic narrators. Although we hypothesised that having higher similarity of autistic traits might result in greater empathy, the participants in our High-AQ Group had a mean AQ of 28.9, whereas the mean AQ score in the clinical autistic population is 35.2 (Ruzich et al., 2015). Furthermore, the AQ is a self-report measure and although it may be used as a screening tool, scoring highly on this measure is not the same as being diagnosed as autistic; for example, people with other conditions such as anxiety and schizophrenia may have high levels of autistic traits (Sasson & Bottema-Beutel, 2022). It must also be emphasised that none of our participants were asked if they had an autistic diagnosis: This was a clear limitation in our study design and future studies should include diagnosed autistic participants.

Contrary to our prediction, our findings showed non-autistic participants having equal ability to name (CogEmp) and share (AffEmp) the emotions of autistic and non-autistic narrators. This could be because participants were only given six options and could have guessed the narrator’s emotion. If guessed correctly, the participant may have felt social pressure to empathise with the narrator, despite not actually feeling the same way. Displaying empathic behaviour is socially desirable and contributes to response bias in self-reported empathy measures (Sassenrath, 2020).

Although no main effect of Narrator-Type was found for CogEmp or AffEmp, we did find main effects of Group. Participants in the High-AQ Group had lower mean CogEmp and AffEmp scores than those in the Low-AQ Group and significantly lower mean AffEmp scores than those in the Medium-AQ Group. Interestingly, these findings were only evident in the empathic measures requiring the participant to label the emotion, whereas both the EmpAcc score measured during the EmpAcc task and the ParInt score measured during the modified emBODY task rated emotional intensity rather than an ability to give emotions perceived labels. Perhaps this is again due to co-occurring alexithymia and difficulty labelling emotions. Santiesteban et al. (2021) reported autistic participants using reduced affective language compared to non-autistic controls. They also found autistic participants exhibiting lower empathy scores compared to non-autistic controls when participants were asked to rate narrators’ affects retrospectively (offline). Conversely, when participants were asked to rate the affective states of narrators continuously while watching the clips (online), they reported no differences between autistic and non-autistic participants’ empathy ratings. In contrast to these findings, McKenzie et al. (2022) found no differences in total CogEmp and AffEmp between autistic and non-autistic participants during the EmpAcc task; however, this study lacked statistical power and so may have failed to detect group differences.

### Strengths and limitations

This study had several strengths. First, the novel design of this study allowed the double empathy problem to be explored in the context of EmpAcc as well as through an embodied felt sense of emotion. Second, unlike previous studies exploring the double empathy problem, our study used dynamic and more ecologically valid stimuli to observe non-autistic participants’ ability to accurately empathise with the emotions of autistic and non-autistic narrators rather than just interpreting mental states or emotion expression recognition. Finally, we used a relatively large sample meaning that the study was adequately powered. Despite the strengths of this study, we acknowledge several limitations. Most critically, we only used participants from the general population instead of including diagnosed autistic participants. This severely limited the inferences that could be drawn and only really tested one side of the double empathy problem, a criticism made by Chown et al. (2020). Furthermore, our study only involved adults from a similar cultural background without intellectual disability. Future studies should include children and adolescents and a range of cultural backgrounds. Moreover, although our study included male and female narrators, all narrators were White, highly educated and from stable socioeconomic backgrounds, it would be beneficial to include narrators from other ethnicities and backgrounds in future studies. Finally, we did not examine individual differences, such as alexithymia, which are known to strongly affect empathic abilities.

In conclusion, this study provides compelling evidence that people from the general population, including those with High-AQ, Medium-AQ and Low-AQ, struggle to accurately empathise with autistic people compared to non-autistic people. The non-autistic participants had significantly lower EmpAcc scores overall when viewing autobiographical accounts of emotional events from autistic narrators compared to non-autistic narrators. They were less able to track the emotions of autistic narrators than non-autistic narrators unless the participant felt the emotion more intensely in the body as was the case with anger and fear. This is a novel finding which supports the double empathy hypothesis and has strong implications for both therapeutic and interpersonal relationships with autistic people. Failure to empathise with autistic people’s emotions could have detrimental effects on their self-esteem, mental health and well-being and how well they are supported. It is, therefore, crucial that awareness of differences in how autistic people communicate and express emotion is emphasised in training of caregivers, educators, healthcare practitioners and therapists. Future research should concentrate on how to increase empathy among the normative population and reduce the responsibility and burden of autistic people having to fit in.

## Supplemental Material

sj-docx-1-aut-10.1177_13623613241252320 – Supplemental material for Do you feel me? Autism, empathic accuracy and the double empathy problem Supplemental material, sj-docx-1-aut-10.1177_13623613241252320 for Do you feel me? Autism, empathic accuracy and the double empathy problem by Rachael TS Cheang, Maya Skjevling, Alexandra IF Blakemore, Veena Kumari and Ignazio Puzzo in Autism

sj-docx-2-aut-10.1177_13623613241252320 – Supplemental material for Do you feel me? Autism, empathic accuracy and the double empathy problem Supplemental material, sj-docx-2-aut-10.1177_13623613241252320 for Do you feel me? Autism, empathic accuracy and the double empathy problem by Rachael TS Cheang, Maya Skjevling, Alexandra IF Blakemore, Veena Kumari and Ignazio Puzzo in Autism

sj-docx-3-aut-10.1177_13623613241252320 – Supplemental material for Do you feel me? Autism, empathic accuracy and the double empathy problem Supplemental material, sj-docx-3-aut-10.1177_13623613241252320 for Do you feel me? Autism, empathic accuracy and the double empathy problem by Rachael TS Cheang, Maya Skjevling, Alexandra IF Blakemore, Veena Kumari and Ignazio Puzzo in Autism
