# Supplementary material for: Do you feel me? Autism, empathic accuracy and the double empathy problem
Source: Autism. 2024 May 17;29(9):2315–27. doi: 10.1177/13623613241252320 (PMC12332230; doi:10.1177/13623613241252320)
Supplement: sj-docx-1-aut-10.1177_13623613241252320 – Supplemental material for Do you feel me? Autism, empathic accuracy and the double empathy problem [file sj-docx-1-aut-10.1177_13623613241252320.docx]

# Positionality Statement

I am an autistic white female researcher diagnosed at the age of 30. Being undiagnosed for the majority of my life, I have struggled with not fitting in and frequently experienced depression and anxiety. My special interest in Buddhism and meditation helped me not only to cultivate self-compassion, but also provided a profound clarity to navigate the complexities of my mind. As a meditation teacher, and through my research on autism, mindfulness, empathy and compassion, I hope to bridge gaps in understanding and empower autistic individuals to feel more understood in a neurodiverse world. As a mother of two autistic children, I am both saddened and motivated by the alarming statistics on suicidality within the autistic community, propelling me to advocate for increased support and greater empathy. I acknowledge that my positionality influences how my research is conducted, the research questions, its outcomes, and the interpretation of the results.
