# Supplementary material for: Do you feel me? Autism, empathic accuracy and the double empathy problem
Source: Autism. 2024 May 17;29(9):2315–27. doi: 10.1177/13623613241252320 (PMC12332230; doi:10.1177/13623613241252320)
Supplement: sj-docx-2-aut-10.1177_13623613241252320 – Supplemental material for Do you feel me? Autism, empathic accuracy and the double empathy problem [file sj-docx-2-aut-10.1177_13623613241252320.docx]

# Supplementary Material

## EmpAcc task construction

Four narrators were recruited through opportunity sampling, one male and one female neurotypical narrator and one male and one female autistic narrator. All narrators were white, three were British and one American. Autistic narrators had a clinical diagnosis of Autism Spectrum Disorder. All narrators completed the AQ to determine the level of their autistic traits, neurotypical narrators were excluded if they had an AQ score >20, autistic narrators were excluded if they had an AQ score <30. The narrators were informed of the aims of the research, their role and the intended audience for their video clips. They were asked to consent to participate in the study and to consent to the use of their videos in this and potential future studies with a similar aim. Each narrator was asked to write a short paragraph about four different autobiographical events where they experienced one of four basic emotions (happy, sad, angry, frightened). They were asked to rate the intensity of the emotion they felt during the event from 1-9. The narrator was then asked to discuss each event with the researcher to elicit the emotion linked to the event. Once the narrator felt that they were re-experiencing the emotion, the researcher commenced filming the narrator for around 1.5-2 minutes describing the event, without making explicit reference to the emotion they felt.

The narrator was filmed, in a room with a plain backdrop, with only the head and shoulders of the narrator in view. After filming had ended, the narrator was asked to watch the clip and rate the intensity of the emotion that they felt whilst speaking about the event on a 9-point scale at 4 time points during the clip. After viewing the video clip, the narrators were further asked to name the emotion that they felt during the filming and indicate on a body map where they felt that emotion in their body and at what intensity. The narrator was given a 10-minute break before repeating the procedure for the next emotion. This allowed residual emotions to reduce in intensity before eliciting the next emotion. Once the narrator had filmed all four emotions, they were asked to film a ‘neutral’ clip, where they had to describe a neutral setting/situation such as their bedroom. The filming took approximately two hours and the narrator was paid a £20 Amazon voucher for their time.

In total 20 clips were filmed, these were arranged into two playlists (A and B). Each playlist included all four narrators, had an autistic narrator and a neurotypical narrator describing each emotion and a neutral event and also one male and one female describing each emotion and a neutral event. The clips were played in a random order for each participant.

Table S1

*Playlist content*

| Playlist A | | | Playlist B | | |
| --- | --- | --- | --- | --- | --- |
| Gender | Narrator-Type | Emotion | Gender | Narrator-Type | Emotion |
| male | autistic | sad | female | neurotypical | angry |
| female | neurotypical | scared | female | autistic | angry |
| male | neurotypical | neutral | female | neurotypical | happy |
| female | autistic | happy | male | autistic | happy |
| female | neurotypical | sad | female | neurotypical | neutral |
| female | autistic | neutral | male | autistic | neutral |
| male | neurotypical | angry | female | autistic | sad |
| female | autistic | scared | male | neurotypical | sad |
| male | autistic | angry | male | neurotypical | scared |
| male | neurotypical | happy | male | autistic | scared |

## Non-significant results

### EmpAcc

EmpAcc was not statistically significantly different for non-autistic narrators (M = 0.86, SD = 0.12) compared to autistic narrators (M = 0.85, SD = 0.11) for anger, *F*(1, 73) = 0.271, *p* = .604, partial η^2^ = .004; for non-autistic narrators (M = 0.89, SD = 0.08) compared to autistic narrators (M = 0.90, SD = 0.09) for fear, *F*(1, 73) = 0.15, *p* = .703, partial η^2^ = .002; or for non-autistic narrators (M = 0.83, SD = 0.10) compared to autistic narrators (M = 0.84, SD = 0.11) for the neutral condition, *F*(1, 73) = 0.286, *p* = .594, partial η^2^ = .008. There was no statistically significant three-way interaction between Narrator-Type, Emotion and Group *F*(5.70, 208.17) = 0.61, *p* < .717, ε = .713, partial η^2^ = .016. There were no statistically significant two-way interactions between: Narrator-Type and Group, *F*(2, 73) = 0.21, *p* = .808, ε = .713, partial η^2^ = .006, or Emotion and Group *F*(5.51, 208.17) = 0.198, *p* = .971, ε = .689, partial η^2^ = .005.

### BodyEmp

Participants across the sample did not differ in BodyEmp when viewing clips of autistic narrators compared to when viewing clips of non-autistic narrators. The main effect of Narrator-TypeBodyEmp *F*(1, 78) = 0.64, *p* = .428, partial η^2^ = .008. There was no significant three-way interaction involving Narrator-Type, Emotion and participant Group *F*(4.91, 163.45) = 0.901, *p* < .469, ε = .524, partial η^2^ = .023 (see Table 2). There were also no significant two-way interactions between: Narrator-Type and Group, *F*(2, 78) = 0.78, *p* = .462, ε = .524, partial η^2^ = .020; Emotion and Group, *F*(4.12, 160.47) = .208, *p* = .937, ε = .514, partial η^2^ = .005; or Narrator-Type and Emotion *F*(2.10, 163.45) = 0.39, *p* = .690, ε = .524, partial η^2^ = .005.

### ParInt

ParInt was not statistically significantly different for non-autistic narrators (M = 0.89, SD = 1.20) compared to autistic narrators (M = 0.97, SD = 1.41) for sadness, *F*(1, 78) = 0.23, *p* = .636, partial η2 = .003; for non-autistic narrators (M = 0.59, SD = 1.09) compared to autistic narrators (M = 0.65, SD = 1.06) for happiness, *F*(1, 78) = 0.26, *p* = .613, partial η2 = .003; or for non-autistic narrators (M = 0.28, SD = 0.58) compared to autistic narrators (M = 0.31, SD = 0.58) for the neutral condition, *F*(1, 78) = 1.29, *p* = .260, partial η2 = .016. There was no statistically significant three-way interaction between Narrator-Type, Emotion and Group *F*(6.02, 234.86) = 0.48, *p* < .825, ε = .753, partial η2 = .012. There were no statistically significant two-way interactions between: Narrator-Type and Group, *F*(2, 78) = 1.09, *p* = .342, ε = .753, partial η2 = .027, or Emotion and Group *F*(6.56, 255.72) = 1.11, *p* = .355, ε = .820, partial η2 = .028.
